# Efficacy of Three Toothpastes in Iron Stain Removal from Primary Teeth

**DOI:** 10.5005/jp-journals-10005-1580

**Published:** 2019

**Authors:** Alireza Heidari, Mehdi Shahrabi, Marzieh S Shahrabi

**Affiliations:** 1–3 Department of Pediatric Dentistry, School of Dentistry, Tehran University of Medical Sciences, Tehran, Iran

**Keywords:** Iron, Primary teeth, Stain, Toothpastes

## Abstract

**Aim:**

This study aimed to assess the iron stain removal efficacy of three toothpastes in extracted primary teeth.

**Materials and methods:**

In this *in vitro* study, 60 extracted sound primary teeth were selected, decoronated at the cementoenamel junction, and their pulp chambers were filled with a composite. The teeth were then immersed in ferrous sulfate solution and brushed 3,000 times in an automatic tooth-brushing machine using Colgate, whitening Crest, and conventional Crest dentifrices. Stain removal was done in four groups using a prophylaxis paste. Digital images were obtained from the teeth before and after brushing with dentifrices and the changes in color parameters were measured using Photoshop and iColor software programs. Changes in color parameters were statistically analyzed using one-way ANOVA while multiple comparisons were done by Tukey's test.

**Results:**

The greatest change in chroma was observed in the prophylaxis group and then in the whitening Crest, Colgate, and conventional Crest (mean values of −65.2, −31.07, −21.27, and −0.73, respectively). Prophylaxis completely removed the stains. The greatest reduction in value occurred in conventional Crest, Colgate, and whitening Crest (−18.07, −12.23, and −0.4, respectively). In the L*a*b* system, the least reductions were noted in the whitening Crest, Colgate, and conventional Crest (mean values of −1.96, −3.92, and −3.37, respectively). Prophylaxis significantly improved tooth brightness (a mean increase of 4.26).

**Conclusion:**

None of the toothpastes in this study were capable of effectively removing iron stains. Crest whitening toothpaste was slightly effective in this regard.

**Clinical significance:**

Iron drops are routinely prescribed for children younger than 2 years of age to prevent iron deficiency and iron deficiency anemia. However, iron stains on teeth are a common concern for many parents. Finding toothpastes with greater efficacy for iron stain removal can help in this respect.

**How to cite this article:**

Heidari A, Shahrabi M, *et al.* Efficacy of Three Toothpastes in Iron Stain Removal from Primary Teeth. Int J Clin Pediatr Dent 2019;12(1):10–14.

## INTRODUCTION

At present, dental esthetics is especially important and many patients present to dental offices complaining of unesthetic and discolored anterior teeth and demand esthetic restorations. In general, two types of tooth discolorations may occur, namely discoloration due to extrinsic factors and congenital/systemic discoloration. To find the etiology of tooth discoloration and proper treatment planning are often challenging for clinicians.

The main reason for tooth discoloration is the accumulation of external stains on the surface or within the pellicles covering the tooth surfaces.^1^ Such stains are formed within the salivary pellicles due to chromogens present in foods and drinks, such as tannins in tea and coffee or habits like smoking.^1,2^ The staining ability of some coloring agents such as black tea and coffee is further enhanced when cationic mouthrinses, such as chlorhexidine or salts of polyvalent metals such as tin and iron are used.^3^ Adsorption and deposition of different agents on the tooth surfaces are responsible for extrinsic staining. Such adsorption occurs due to electrostatic forces, hydration, hydrogen bonds, and hydrophilic reactions. However, the exact mechanism of adhesion of these stains to tooth surfaces is yet to be clearly understood.^4^

Iron drops are routinely prescribed for children younger than 2 years of age to prevent iron deficiency and iron deficiency anemia. Iron drops have several advantages and improve iron intake in children. However, they can cause black discoloration of teeth. Such discoloration would have an adverse effect on the personality and mood of children and may affect their ability to communicate with their peers and may result in their isolation and family concerns. At present, a low speed hand piece with a rotary brush and prophylactic paste are used for iron stain removal. This is done in dental clinics and requires the cooperation of children. Lack of cooperation of children may complicate the process of stain removal. Whitening toothpastes are used at home for removal of iron stains from teeth in children. This study aimed to assess the iron stain removal efficacy of three toothpastes in extracted primary teeth.

## MATERIALS AND METHODS

This *in vitro* experimental study was conducted on 60 sound primary anterior teeth with no caries, restorations, developmental defects, enamel cracks, or external discoloration. The teeth had been extracted due to orthodontic reasons. Using randomization blocks, the teeth were randomly divided into four groups. Soft tissue residues were manually removed from the teeth surfaces and the teeth were immersed in 5.25% sodium hypochlorite solution (Shamin Chemical, Tehran, Iran) for 30 minutes. The teeth were then stored in sterile 0.9% sodium saline solution (DaruPakhsh, Tehran, Iran) at room temperature until the experiment was complete. In the next step, the teeth were cut at the cementoenamel junction (CEJ) and the pulp chamber was evacuated. The CEJ area and the pulp chamber were filled with flowable composite resin. The teeth were first etched for 30 seconds, rinsed and the bonding agent was applied and light cured for 10 seconds. The flowable composite was then injected to fill up the area. The teeth were mounted on a specific mold containing an auto polymerizing acrylic resin. The teeth were then immersed in ferrous sulfate solution (iron drop) for 30 hours. The teeth were photographed by a digital camera (Canon). The method and angulation of lighting and photography were the same for all specimens. The digital camera was fixed to a tripod and the tripod was also fixed to the ground. The camera was adjusted at 20 cm distance from the specimen surface. Mounted teeth were photographed against a green background. Colgate children's toothpaste was used for teeth in group A, Crest adults’ toothpaste was used in group B, Crest whitening toothpaste was used in group C, and group D teeth were subjected to prophylaxis using a prophylactic rotary brush and paste. The afore-mentioned toothpastes and 45 GUM 411 toothbrushes were obtained from the official representatives.

The teeth were brushed using an automatic tooth-brushing machine 3,000 times. For this purpose, eight toothbrushes were simultaneously installed in V8 cross-brushing machine and the specimens were fixed beneath the toothbrushes in their respective location. Next, 25 g of the toothpaste was dissolved in 100 mL of water and a solution of each toothpaste was prepared. A separate toothbrush was used for each tooth. A total of 15 teeth were brushed with a low speed hand piece and prophylactic paste for 10 seconds. Digital photographs were taken again at the same conditions from the specimens. Images were transferred to a computer and evaluated using Adobe Photoshop CS5 and iColor software programs. For this purpose, the L*, a*, and b* parameters were determined for specimens in Photoshop software, while the *L* (value), *C* (chroma), and *H* (hue) parameters were calculated using the iColor software.

The range of changes for the L*, a* and b* parameters in Adobe Photoshop 7.0 software varies from 0 to 255. However, in the CIE L*a*b* system, the L* parameter varies from 0 to 100 and a* and b* range from −120 to +120. Thus, considering the different ranges of change in L*, a*, and b* values in Photoshop software with the CIE L*a*b* parameters, the following equations were used for conversion of values to the CIE system:


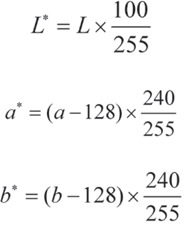


Δ*E* was then calculated using the equation below:


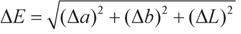


The L*, a*, and b* parameters were calculated in Photoshop software before and after the use of toothpastes, while the LCH parameters were calculated in the iColor software before and after the intervention. The differences in the treatment groups in terms of changes in the L*, a*, and b* parameters in Photoshop software and LCH parameters in iColor software were analyzed using one-way ANOVA. In cases for which the results of ANOVA became significant, pairwise comparison of groups was carried out using Tukey's multiple comparison test.

## RESULTS

In group A, using Colgate children's toothpaste, the L* parameter, the b* parameter, and the a* parameter changed by 3.92 ± 1.99, 8.09 ± 4.72, and 22.78 ± 3.51, respectively, after the intervention (tooth brushing with the toothpaste). In group B, using the Crest adults’ toothpaste, the L* parameter, the b* parameter, and the a* parameter changed by 3.37 ± 2.33, 4.27 ± 4.95, and 20.33 ± 4.43, respectively, after the intervention (tooth brushing with the toothpaste). In group C, using the Crest whitening toothpaste, the L* parameter, the b* parameter, and the a* parameter changed by 1.96 ± 2.14, 9.29 ± 4.31, and 23.22 ± 3.81, respectively, after the intervention (tooth brushing with the toothpaste). In group 4 (prophylaxis), the L* parameter, the b* parameter, and the a* parameter changed by 4.26 ± 2.6, 12.11 ± 10.23, and 22.53 ± 8.02 after the intervention, respectively. The Δ*E* of teeth in the four groups A, B, C, and D was found to be 24.83 ± 4.55, 21.63 ± 4.79, 25.44 ± 4.29, and 27.67 ± 8.69, respectively, using the Photoshop software (positive values indicate the increase in parameters while negative values indicate reduction in parameters after tooth brushing) (Table 1).

One-way ANOVA revealed significant differences in the range of changes in L* (*p* < 0.0001), b* (*p* < 0.02), and Δ*E* (*p* < 0.05) among different toothpaste groups; however, no significant difference was noted among groups in terms of the range of changes in a* parameter (*p* = 0.45). Considering the significant results of one-way ANOVA for L*, b*, and Δ*E*, pairwise comparison of groups in this regard was carried out using Tukey's multiple comparison test. The results showed that the Colgate children's toothpaste and Crest adults’ toothpaste (*p* = 0.91), and also Colgate children's toothpaste and Crest whitening toothpaste (*p* = 0.33) were not significantly different in terms of changes in the L* parameter after the intervention. However, group A (Colgate children's toothpaste) and group D (*p* < 0.0001), group B (Crest adults’ toothpaste) and group D (*p* < 0.0001), and also group C (Crest whitening toothpaste) and group D (*p* < 0.0001) showed significant differences in this regard.

Regarding the b* color parameter, significant differences were not found between group A (Colgate children's toothpaste) and group B (Crest adults’ toothpaste) (*p* = 0.38), group A (Colgate children's toothpaste) and group C (Crest whitening toothpaste) (*p* = 0.96), group A and group D (*p* = 0.34), group B and group C (*p* = 0.16), and group C and group D (*p* = 0.64). However, the difference between group D and group B (Crest adults’) in the b* parameter was found to be statistically significant after the intervention (*p* < 0.009).

Comparison of Δ*E* revealed significant differences only between groups B and D (*p* < 0.03) and no other significant differences were found (*p* = 0.45 for groups A and B, *p* = 0.99 for groups A and C, *p* = 0.55 for groups A and D, *p* = 0.29 for groups B and C and *p* = 0.73 for groups C and D).

**Table 1:** The mean and standard deviation of changes in the L*, a*, and b* parameters in the Photoshop software after toothbrushing with different toothpastes (test groups) and prophylaxis (control)

*Group*	*Parameter*	*Mean*	*SD*	*Minimum*	*Maximum*	*Number*
Toothpaste Colgate children's	L*	−3	1.99	−6.67	0.39	15
	b*	−8.09	4.72	−16.94	1.88	15
	a*	−22.78	3.51	−29.18	−16.0	15
	Δ*E*	24.83	4.55	16.12	33.77	15
Crest adults’	L*	−3.37	2.33	−6.67	0	15
	b*	−4.27	4.95	−16.0	4.71	15
	a*	−20.33	4.43	−27.29	−12.24	15
	Δ*E*	21.63	4.79	13.32	29.75	15
Crest whitening	L*	−1.96	2.14	−5.1	2.75	15
	b*	−9.29	4.31	−15.06	1.88	15
	a*	−23.22	3.81	−31.06	−16.0	15
	Δ*E*	25.44	4.29	17.13	32.6	15
Prophylaxis	L*	4.26	2.6	−1.18	8.24	15
	b*	−12.11	10.23	−28.24	16.94	15
	a*	−22.53	8.02	−32.94	−7.53	15
	Δ*E*	27.67	8.69	14.29	43.45	15

The iColor software revealed that Colgate children's toothpaste changed hue by 2.13 ± 4.26, chroma by 21.27 ± 15.46, and value by 12.33 ± 7.84 units after the intervention. Crest adults’ toothpaste changed hue by 2.53 ± 2.85, chroma by 19.83 ± 0.73, and value by 18.07 ± 10.23 units after the intervention. Crest whitening toothpaste changed hue by 3.28 ± 0.07, chroma by 31.07 ± 18.96, and value by 15.38 ± 0.4 units after the intervention. Prophylaxis changed hue by 3.59 ± 1.8, chroma by 65.2 ± 16.88, and value by 34.07 ± 13.72 units (Table 2) (positive values indicate an increase in parameters, while negative values indicate a reduction in parameters).

One-way ANOVA showed that the four groups were not significantly different in terms of changes in hue (*p* = 0.25) but they showed significant differences in terms of changes in chroma (*p* < 0.0001) and value (*p* < 0.0001).

Considering the significant differences in chroma and value, a pairwise comparison of groups was done using Tukey's test. In terms of chroma, the differences between Colgate and Crest adults’ (*p* < 0.01), Colgate and group D (*p* < 0.0001), Crest adults’ and Crest whitening (*p* < 0.0001), Crest adults’ and group D (*p* < 0.0001) and Crest whitening and group D (*p* < 0.0001) were statistically significant. However, no significant difference was found between Colgate children's toothpaste and Crest whitening toothpaste (*p* = 0.44) in terms of chroma in the iColor software.

In terms of value, the difference between Colgate and Crest adults’ (*p* = 0.57) was not significant but the differences between Colgate children's toothpaste and Crest whitening (*p* = 0.05), Colgate children's and group D (*p* < 0.0001), Crest adults’ and Crest whitening (*p* < 0.001), Crest adults’ and group D (*p* < 0.0001) and Crest whitening and group D (*p* < 0.0001) were statistically significant.

## DISCUSSION

This study showed significant differences in the range of changes in the L*, b* and Δ*E* color parameters after tooth brushing with different toothpastes; however, the difference in the range of changes in the a* parameter was not significant among groups. The greatest Δ*E* was noted in group D (prophylaxis) followed by group C (Crest whitening), group A (Colgate children's), and group B (Crest adults’) with a mean value of 27.67, 25.44, 24.83, and 21.63, respectively. In other words, all the interventions in the current study caused color changes in teeth but the color changes were not necessarily towards lightening of the color shade. The difference between group D and group B in terms of mean Δ*E* was significant but the remaining groups showed no significant difference in this regard.

The greatest change in chroma occurred in group D followed by group C, group A, and group B (65.2, 31.07, 21.27, and 0.73, respectively). In total, the teeth became slightly lighter but prophylaxis completely removed the stains.

The greatest reduction in value was noted in group B, group A, and group C (18.07, 12.33, and 0.4, respectively). This means that the teeth slightly lost value but did not appear darker. Crest whitening toothpaste had the greatest efficacy in this regard and reduction in value due to the application of this toothpaste being negligible. This value significantly increased following prophylaxis and the teeth became lighter (mean value of 34.07). The results of the L*a*b* system also confirmed this finding because the smallest reduction in the L* parameter (lightness) was found in group C (Crest whitening with a mean value of 1.96) followed by Colgate children's and Crest adults’ toothpastes (3.92 and 3.37, respectively). Prophylaxis significantly lightened the teeth (a mean value of 4.26).

Color has three parameters hue, value, and chroma. Hue is the perception of observer from the color and depends on the different wavelengths of light beams that reach the eyes. Value is the achromatic dimension of color and indicates its lightness/darkness. The higher the value, the lighter the color and the lower the value, the darker the color. Chroma is the intensity of color. The greater the chroma, the richer the color. In total, Crest whitening toothpaste slightly removed the iron stains but Crest adults’ and Colgate children's toothpastes did not cause a significant difference in this regard. Prophylaxis successfully removed all stains.

Esfahanizadeh and Ghayumi showed that Crest whitening toothpaste was more effective than Pooneh in stain removal. In our study, Crest whitening toothpaste caused the greatest color change and was found to be more efficient that other toothpastes. In a study by Sharif et al., in 2000, toothpastes containing sodium tripolyphosphate and sodium lauryl sulfate were more effective in stain removal than toothpastes free from sodium tripolyphosphate.^5^ Ayad et al. evaluated the stain removal efficacy of three toothpastes from composite restorations and reported that toothpastes containing polymeric phosphates (tetrasodium pyrophosphate and sodium tripolyphosphate) were more effective than toothpastes devoid of these compounds.^6^ Aside from sodium tripolyphosphate, tetra sodium pyrophosphate is also effective as an anti-plaque agent for this purpose.^6^ In the study, by Esfahanizadeh and Ghayumi, Crest whitening toothpaste was found to be more effective than Pooneh in terms of stain removal because Crest whitening toothpaste contains tetrasodium pyrophosphate as an anti-plaque agent. Thus, this explanation also applies to the current study. Polyvinylpyrrolidone (PVP) is another compound that bonds to tea and chlorhexidine stains and removes them from the tooth surface. A previous study assessed the efficacy of an experimental toothpaste containing PVP and reported that it was more effective for prevention of stains than commercial toothpastes and water.^7^

**Table 2:** The mean and SD of differences in hue, chroma, and value in the iColor software after toothbrushing with different toothpastes (test groups) or prophylaxis (control group)

*Group*	*Parameter*	*Mean*	*SD*	*Minimum*	*Maximum*	*Number*
Toothpaste Colgate children's	Hue	−2.13	4.26	−8.0	9.0	15
	Chroma	−21.27	15.46	−43.0	9.0	15
	Value	−12.33	7.84	−26.0	0	15
Crest adults’	Hue	−2.53	2.85	−7.0	2.0	15
	Chroma	−0.73	19.83	−25.0	59.0	15
	Value	−18.07	10.23	−35.0	−6.0	15
Crest’ whitening	Hue	−0.07	3.28	−6.0	6.0	15
	Chroma	−31.07	18.96	−58.0	8.0	15
	Value	−0.4	15.38	−26.0	37.0	15
Prophylaxis	Hue	−1.8	3.59	−8.0	5.0	15
	Chroma	−65.2	16.88	−98.0	−46.0	15
	Value	34.07	13.72	13.0	59.0	15

Pontefract et al. evaluated the efficacy of Aquafresh experimental toothpaste for stain removal in comparison with commercial toothpastes and water.^8^ The superior efficacy of Aquafresh reported in their study was attributed to the presence of sodium tripolyphosphate, sodium lauryl sulfate, and PVP whitening agent in its formulation.

Moran et al. found no significant difference between an experimental toothpaste containing sodium tripolyphosphate and toothpastes devoid of this compound.^9^ They explained that a combination of physical methods such as tooth brushing with abrasive materials and chemicals present in the formulation of toothpastes decreases dental stains. Reduction in the intensity of stains may be due to the thinning of the stain layer due to the abrasive effect of tooth brushing with toothpastes, and color of stains turns from brown to yellow upon exposure to chemicals present in the formulation of toothpastes.

In a study on toothpastes containing whitening agents such as sodium tripolyphosphate, sodium lauryl sulfate, and PVP, toothpastes containing these compounds were found to be more effective in the removal of external stains than other toothpastes. In a study by Esfahanizadeh et al., although both Crest whitening and Pooneh toothpastes contained these three compounds, Crest whitening toothpaste was found to be more efficient than Pooneh. This controversy may be due to the presence of tetra sodium pyrophosphate as an antiplaque agent in the composition of Crest and the small size of tripolyphosphate particles present in Crest toothpaste.

Golpas and Hagh et al. compared Iranian and foreign made whitening toothpastes with a conventional toothpaste and reported that all toothpastes were effective in decreasing the stains. However, use of different indexes in recent studies makes accurate comparison of results difficult because the afore-mentioned study was a clinical study and the intensity of stains was measured using Lobene's index. Whereas the current study had an *in vitro* design and measured L*a*b* color parameters. In the current study, Crest whitening toothpaste slightly removed the iron stains (by decreasing chroma).

Sharma et al. reported that Colgate and Aquafresh whitening and fluoridated toothpastes were more effective in stain removal than Crest regular toothpaste.^10^ In our study, Crest whitening and Colgate children's toothpastes were more effective in improving tooth color and removing stains than Crest adults’ toothpaste. Yankell et al. reported that Aquafresh whitening toothpaste and Colgate anti-calculus toothpaste were more efficient in decreasing external stains compared to Crest regular toothpaste.^11^ Another study showed that whitening toothpastes were more capable of decreasing external stains compared to regular toothpastes.^12^ Moreover, Colgate toothpaste is capable of removing more stains than Crest toothpaste after 3 and 6 months.^13^

Abrasive agents like silica incorporated into the formulations of toothpastes physically remove stains. At present, silica is incorporated into the formulations of most toothpastes. Thus, such efficacy may be expected even from regular (non-whitening) toothpastes. However, in the current study, Colgate children's and Crest regular toothpastes showed no efficacy for iron stain removal, which may be due to the nature of stains. Addition of chemicals to whitening toothpastes enables the toothpastes to exert their cleaning effect even on areas that are hard to reach by abrasives. The use of toothpastes and toothbrushes enables benefitting from both the abrasiveness of toothbrushes and the physicochemical properties of toothpastes in stain removal. On the other hand, the combination of abrasive and chemical agents in toothpastes changes the nature of stains. Also, the decreased intensity of stains may be due to the narrowing of the stain layer due to the abrasive action of toothbrush and toothpastes or changed color parameters.

Most previous studies have only used indexes to measure the intensity and extensiveness of stains and digital equipment and software programs have been less commonly used for this purpose. Use of these technologies in the current study increased the accuracy of results. However, the study of specimens in the clinical setting would be more advantageous. Moreover, since the current study was conducted on primary teeth, comparison and generalization of results to permanent teeth would be difficult.

## CONCLUSION

None of the toothpastes in this study were capable of effectively removing iron stains. Crest whitening toothpaste was slightly effective in this regard. Thus, none of these toothpastes are recommended for iron stain removal following iron drop consumption.

## CLINICAL SIGNIFICANCE

Iron drops are routinely prescribed for children younger than 2 years of age to prevent iron deficiency and iron deficiency anemia. However, iron stains on teeth are a common concern for many parents. Finding toothpastes with greater efficacy for iron stain removal can help in this respect. This study showed that none of the toothpastes in this study were capable of effectively removing iron stains. Crest whitening toothpaste was slightly effective in this regard.
